# Adjunctive Volasertib in Patients With Acute Myeloid Leukemia not Eligible for Standard Induction Therapy: A Randomized, Phase 3 Trial

**DOI:** 10.1097/HS9.0000000000000617

**Published:** 2021-08-02

**Authors:** Hartmut Döhner, Argiris Symeonidis, Dries Deeren, Judit Demeter, Miguel A. Sanz, Achilles Anagnostopoulos, Jordi Esteve, Walter Fiedler, Kimmo Porkka, Hee-Je Kim, Je-Hwan Lee, Kensuke Usuki, Stefano D'Ardia, Chul Won Jung, Olga Salamero, Heinz-August Horst, Christian Recher, Philippe Rousselot, Irwindeep Sandhu, Koen Theunissen, Felicitas Thol, Konstanze Döhner, Veronica Teleanu, Daniel J. DeAngelo, Tomoki Naoe, Mikkael A. Sekeres, Valerie Belsack, Miaomiao Ge, Tillmann Taube, Oliver G. Ottmann

**Affiliations:** 1 Department of Internal Medicine III, Ulm University Hospital, Ulm, Germany; 2 Hematology Division, University Hospital, University of Patras Medical School, Patras, Greece; 3 AZ Delta, Roeselare, Belgium; 4 Semmelweis University, Budapest, Hungary; 5 Department of Hematology, University Hospital La Fe, Valencia, Spain; 6 Hematology Department, General Hospital G. Papanikolaou, Thessaloniki, Greece; 7 Hospital Clinic de Barcelona, IDIBAPS, Barcelona, Spain; 8 Department of Medicine II, University Medical Center, Hamburg-Eppendorf, Hamburg, Germany; 9 Department of Hematology, Helsinki University Hospital Cancer Center, Helsinki, Finland; 10 Catholic Hematology Hospital, Seoul St. Mary’s Hospital, College of Medicine, The Catholic University of Korea, Seoul, South Korea; 11 Asan Medical Center, University of Ulsan College of Medicine, Seoul, South Korea; 12 NTT Medical Center Tokyo, Japan; 13 A.O. Città della Salute e della Scienza, Torino, Italy; 14 Samsung Medical Center, Seoul, South Korea; 15 Hospital Vall d’Hebron/VHIO/UAB-Medicine, Barcelona, Spain; 16 UKSH Campus Kiel, Kiel, Germany; 17 Centre Hospitalier Universitaire de Toulouse, IUCT-Oncopole, Université Paul Sabatier Toulouse 3, Toulouse, France; 18 Centre Hospitalier de Versailles, University Versailles Saint-Quentin and Paris Saclay, Le Chesnay, France; 19 Division of Hematology, Department of Medicine, University of Alberta, Edmonton, AB, Canada; 20 Jessa Ziekenhuis, Hasselt, Belgium; 21 Medizinische Hochschule Hannover, Germany; 22 Dana-Farber Cancer Institute, Boston, Massachusetts, USA; 23 National Hospital Organization Nagoya Medical Center, Nagoya, Japan; 24 Cleveland Clinic Taussig Cancer Institute, Cleveland, Ohio, USA; 25 SCS Boehringer Ingelheim Comm.V, Brussels, Belgium; 26 Boehringer Ingelheim Pharmaceuticals, Inc., Ridgefield, Connecticut, USA; 27 Boehringer Ingelheim International GmbH, Biberach, Germany; 28 Goethe University, Frankfurt/Main, Germany

## Abstract

In this phase 3 trial, older patients with acute myeloid leukemia ineligible for intensive chemotherapy were randomized 2:1 to receive the polo-like kinase inhibitor, volasertib (V; 350 mg intravenous on days 1 and 15 in 4-wk cycles), combined with low-dose cytarabine (LDAC; 20 mg subcutaneous, twice daily, days 1–10; n = 444), or LDAC plus placebo (P; n = 222). Primary endpoint was objective response rate (ORR); key secondary endpoint was overall survival (OS). Primary ORR analysis at recruitment completion included patients randomized ≥5 months beforehand; ORR was 25.2% for V+LDAC and 16.8% for P+LDAC (n = 371; odds ratio 1.66 [95% confidence interval (CI), 0.95–2.89]; *P* = 0.071). At final analysis (≥574 OS events), median OS was 5.6 months for V+LDAC and 6.5 months for P+LDAC (n = 666; hazard ratio 0.97 [95% CI, 0.8–1.2]; *P* = 0.757). The most common adverse events (AEs) were infections/infestations (grouped term; V+LDAC, 81.3%; P+LDAC, 63.5%) and febrile neutropenia (V+LDAC, 60.4%; P+LDAC, 29.3%). Fatal AEs occurred in 31.2% with V+LDAC versus 18.0% with P+LDAC, most commonly infections/infestations (V+LDAC, 17.1%; P+LDAC, 6.3%). Lack of OS benefit with V+LDAC versus P+LDAC may reflect increased early mortality with V+LDAC from myelosuppression and infections.

## Introduction

While acute myeloid leukemia (AML) affects people of all ages, the majority of patients are of advanced age, with a median age at diagnosis of approximately 70 years in developed countries.^1,2^ Thus, the incidence of AML is rising, at least in part, as a result of the aging population.^2^

Older AML patients are less likely than younger patients to achieve a complete remission (CR) with standard therapy and tend to have comorbidities that prevent them from receiving intensive chemotherapy.^3^ For these patients, low-intensity therapies, such as subcutaneous administration of low-dose cytarabine (LDAC), are considered better options. As a result, LDAC has become a recommended therapy, and an established comparator and combination partner for investigational drugs, before the introduction of hypomethylating agents.^4,5^

Polo-like kinase 1 (Plk1) is a key regulator of mitosis, and its overexpression has been linked with poor prognosis in human cancer.^6^ Inhibition of Plk1 in vitro was found to block proliferation of leukemic cell lines, and to reduce the clonogenic potential of cell lines derived from patients with leukemia.^7^ Volasertib is a low-molecular-weight, adenosine triphosphate-competitive kinase inhibitor that potently inhibits Plk1, as well as the 2 closely related kinases, Plk2 and Plk3. In a previous study, volasertib treatment reduced tumor growth in colon and lung xenograft models, and increased apoptosis in samples derived from HCT 116 tumor-bearing nude mice.^8^ Volasertib has also shown robust antitumor activity in a xenograft model of AML; nude mice with established AML tumors treated with volasertib for 4 weeks experienced marked tumor regression and tolerated treatment well.^9^

In an open-label, randomized phase 2 trial, conducted in previously untreated AML patients aged ≥65 years who were ineligible for intensive therapy, objective response rates (ORRs; CR or CR with incomplete blood count recovery [CRi]) and overall survival (OS) favored volasertib in combination with LDAC (V+LDAC) over LDAC monotherapy (ORR: 31% versus 13%, odds ratio [OR] 2.91, *P =* 0.052; median OS 8.0 versus 5.2 months, hazard ratio [HR] 0.63 [95% confidence interval (CI), 0.40–1.00]; *P =* 0.047). There was an increase in nonhematologic adverse events (AEs) with V+LDAC compared with LDAC; the AEs with the most pronounced increase in frequency included gastrointestinal AEs grade 3 (21% versus 7%), febrile neutropenia grade 3 (38% versus 7%), and infections grade 3 (38% versus 7%). However, these AEs were clinically manageable.^10^ The current phase 3 study was conducted to confirm the results from the previous phase 2 study of the V+LDAC regimen for older AML patients who are unable to receive intensive therapies.

## Materials and methods

### Patients and study design

This was a prospective, randomized, double-blind, placebo-controlled study (NCT01721876) of V+LDAC compared with placebo + LDAC (P+LDAC). Eligible patients were aged ≥65 years, had previously untreated (except for hydroxyurea) AML (confirmed according to World Health Organization criteria^11^), and an Eastern Cooperative Oncology Group Performance Status (ECOG PS) ≤2. Patients were required to be ineligible for intensive remission-induction therapy, based on documented disease and patient characteristics such as high-risk cytogenetics, secondary AML, and comorbidity. Exclusion criteria included: prior or concomitant treatment for AML (prior treatment for myelodysplastic syndrome was allowed); acute promyelocytic leukemia; clinical signs of leukemic central nervous system involvement; clinically relevant QT prolongation (>470 ms); and inadequate organ function (bilirubin >3× upper limit of normal and/or creatinine clearance <30 mL/min).

Eligible patients were randomized in a 2:1 ratio to receive V+LDAC or P+LDAC via an interactive voice/web response system, stratified according to ECOG PS (0–1 versus 2) and type of leukemia (de novo versus secondary). LDAC was administered subcutaneously at a dose of 20 mg twice daily on days 1–10 of each 4-week cycle, either at the investigative site or at the patient’s home, and either volasertib (350 mg) or placebo was added as a 1-hour intravenous infusion on days 1 and 15. Repeated cycles of treatment (with no limit to the number) were administered until disease progression or relapse, according to protocol-defined criteria for treatment continuation and unless the patient or investigator requested treatment discontinuation. If, at the end of each treatment cycle, criteria to continue treatment were not yet met, or if determined necessary by the investigator, subsequent cycles could be delayed for an unrestricted length of time. Dose reductions of volasertib or placebo were allowed in 50-mg decrements, to a minimum of 200 mg. Given the myelosuppressive effects of both volasertib and LDAC, anti-infective prophylaxis and/or growth factors such as granulocyte colony-stimulating factor could be administered according to local guidelines and standards.

The trial was performed in accordance with the Declaration of Helsinki, the International Conference on Harmonisation Guideline for Good Clinical Practice, and applicable specific requirements, and with the approval of the respective institutional review boards/independent ethics committees at each center. All patients provided written informed consent.

### Study endpoints and assessments

The primary endpoint was ORR, as determined by the central, blinded review of bone marrow samples and the investigator’s assessment (evaluation of peripheral blood and physical examination). Bone marrow examination for response assessment was carried out at the end of every second cycle, or as soon as possible if disease progression was suspected. CR and CRi were defined according to European Leukemia Net (ELN) recommendations,^5^ and an additional criterion for CR was red blood cell transfusion independence within 7 days before response assessment. The key secondary endpoint was OS, defined as the time interval from the date of randomization to the date of death.

Two analyses were planned according to the study protocol. The primary analysis was performed shortly after completion of patient recruitment and assessed the primary efficacy endpoint, ORR, using efficacy data from the subset of patients randomized ≥5 months before the cutoff date, including those without response data. Analysis of OS at the primary analysis was descriptive and exploratory. The final analysis to assess the key secondary endpoint, OS, included all randomized patients and was carried out after at least 574 OS events had occurred.

Safety was assessed by determining the incidence and intensity of AEs, defined using the Common Terminology Criteria for Adverse Events (version 3.0), and changes in laboratory assessments and electrocardiograms. Safety evaluations of the treated populations (all randomized patients who received at least one dose of trial medication) were conducted at both the primary and final analyses.

An independent Data Monitoring Committee periodically reviewed unblinded results to monitor the conduct of the trial, ensure patient safety, and maintain the integrity of the data.

### Statistical considerations

It was estimated that approximately 371 patients should be included in the primary analysis of ORR, providing 90% power to detect an OR of 2.85 (based on the phase 2 study^10^ and a phase 3 study of decitabine for elderly AML patients)^12^ using a 2-sided test and an alpha level of 0.05. A final planned sample size of 660 patients was selected to allow collection of an expected 574 OS events, assuming a dropout rate of ~10%.

The Cochran–Mantel–Haenszel test (adjusting for the 2 stratification factors used for randomization) was used to compare ORR between treatment groups, based on a 2-sided alpha-level of 0.05. Mantel–Haenszel estimates for OR and 95% CI were calculated.

For OS, Kaplan–Meier estimates were calculated for both arms. A log-rank test was carried out, stratified by the same 2 factors used for randomization. A stratified Cox proportional hazards model was used to estimate the HR between arms.

An unplanned, exploratory, post hoc analysis was conducted to better understand the difference between the phase 2 and phase 3 results, and to examine possible reasons for the different outcomes observed in this phase 3 trial (see Supplemental Digital Methods, http://links.lww.com/HS/A177).

## Results

### Patients and treatment

**Figure 1.**
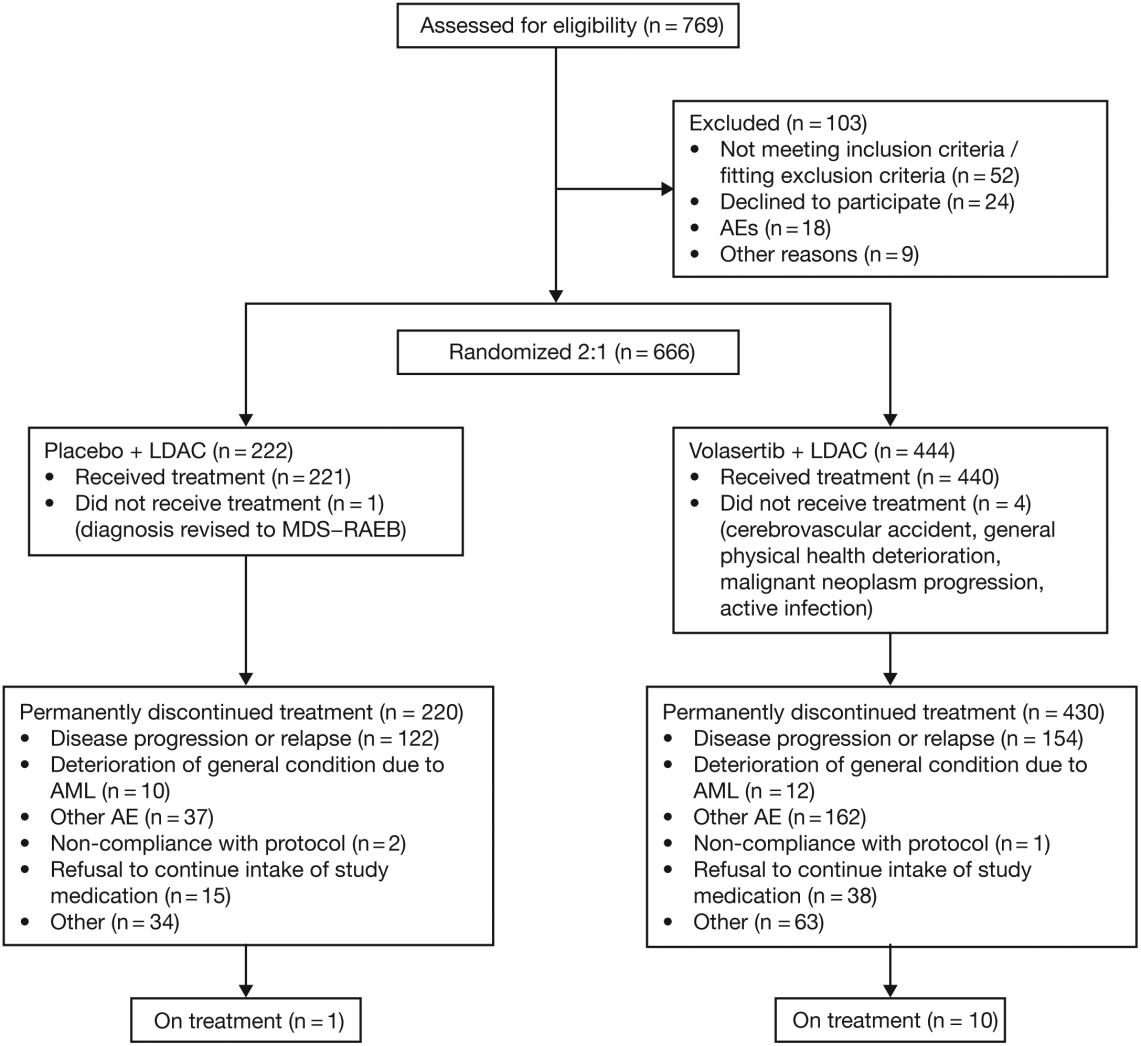
**Disposition of patients included in the final analysis.** AEs = adverse events; AML = acute myeloid leukemia; LDAC = low-dose cytarabine; MDS = myelodysplastic syndrome; RAEB = refractory anemia with excess blasts.

From February 25, 2013, to November 12, 2014, 769 patients were screened at 122 centers in 25 countries, and 666 patients were subsequently randomized (V+LDAC, n = 444; P+LDAC, n = 222). Of these, 661 patients received the study medication (V+LDAC, n = 440; P+LDAC, n = 221) (Figure 1). Patient demographics and baseline disease characteristics were generally balanced between treatment arms (Table 1). The most frequently documented medical reason for ineligibility for intensive remission-induction therapy was age (97.4%), followed by comorbidities (47.3%), most commonly cardiac disorders (20.7%).

**Table 1:** Baseline Patient Demographics and Disease Characteristics

Characteristic	Primary Efficacy Analysis Set		Final Analysis Set	
	P+LDAC (n = 125)	V+LDAC (n = 246)	P+LDAC (n = 222)	V+LDAC (n = 444)
Sex, n (%)				
Male	75 (60.0)	140 (56.9)	135 (60.8)	241 (54.3)
Female	50 (40.0)	106 (43.1)	87 (39.2)	203 (45.7)
Race, n (%)				
White	88 (70.4)	181 (73.6)	158 (71.2)	328 (73.9)
Asian	21 (16.8)	39 (15.9)	39 (17.6)	74 (16.7)
Other/missing	16 (12.8)	26 (10.6)	25 (11.3)	42 (9.5)
Age, median (min–max)	75.0 (65–85)	75.0 (65–93)	76.0 (65–88)	75.0 (65–93)
ECOG PS, n (%)				
0	27 (21.6)	48 (19.5)	53 (23.9)	100 (22.5)
1	65 (52.0)	136 (55.3)	117 (52.7)	241 (54.3)
2	33 (26.4)	62 (25.2)	52 (23.4)	103 (23.2)
WBC count/nL, n (%)				
<10/nL	86 (68.8)	173 (70.3)	149 (67.1)	310 (69.8)
≥10/nL and <50/nL	36 (28.8)	52 (21.1)	62 (27.9)	104 (23.4)
≥50/nL	3 (2.4)	21 (8.5)	11 (5.0)	30 (6.8)
Type of AML, n (%)				
De novo	64 (51.2)	130 (52.8)	114 (51.4)	230 (51.8)
Secondary AML	61 (48.8)	116 (47.2)	108 (48.6)	214 (48.2)
Preceding MDS	45 (36.0)	83 (33.7)	77 (34.7)	162 (36.5)
Preceding MPS	8 (6.4)	17 (6.9)	18 (8.1)	28 (6.3)
Therapy-related^*a*^	8 (6.4)	16 (6.5)	12 (5.4)	24 (5.4)
Other	3 (2.4)	11 (4.5)	10 (4.5)	17 (3.8)
2010 ELN genetic group, n (%)				
Favorable	13 (10.4)	28 (11.4)	21 (9.5)	47 (10.6)
Intermediate I	38 (30.4)	80 (32.5)	71 (32.0)	144 (32.4)
Intermediate II	33 (26.4)	42 (17.1)	46 (20.7)	75 (16.9)
Adverse	36 (28.8)	82 (33.3)	70 (31.5)	142 (32.0)
Missing	5 (4.0)	14 (5.7)	14 (6.3)	36 (8.1)
Mutation types, n (%)				
*NPM1*	16 (12.8)	35 (14.2)	29 (13.1)	68 (15.3)
*FLT3* ITD	6 (4.8)	13 (5.3)	8 (3.6)	22 (5.0)

^*a*^Prior therapy with alkylating agents or topoisomerase II inhibitors.

AML = acute myeloid leukemia; ECOG PS = Eastern Cooperative Oncology Group Performance Status; ELN = European LeukemiaNet; ITD = internal tandem duplication; MDS = myelodysplastic syndrome; MPS = myeloproliferative syndrome; P+LDAC = placebo plus low-dose cytarabine; V+LDAC = volasertib plus low-dose cytarabine; WBC = white blood cell.

Data cutoff for the primary analysis was August 12, 2014; 371 patients had been assessed for the primary efficacy endpoint, ORR (randomized ≥5 mo before data cutoff; V+LDAC, n = 246; P+LDAC, n = 125), and 533 patients had been assessed for safety (received treatment; V+LDAC, n = 356; P+LDAC, n = 177). On December 18, 2014, based on the results of the primary analysis, blinding was suspended for all patients receiving ongoing treatment. Placebo administration was discontinued and the decision whether to continue patients on unblinded study treatment was taken by the investigators, based on individual benefit–risk evaluations and patient informed re-consent.

The subsequent final analysis (June 1, 2017) included all 666 randomized patients for efficacy analyses and all 661 treated patients for safety analyses. The final analysis was exploratory and descriptive, because potential bias was introduced by the unblinding after the primary analysis.

At both the primary and final analyses, the mean number of initiated treatment cycles was higher in the P+LDAC arm versus the V+LDAC arm (3.6 versus 2.8 and 5.1 versus 4.4 cycles, respectively). At both analyses, the median number of treatment cycles initiated was 2.0 for both the P+LDAC and V+LDAC arms (range 1–16 and 1–14 cycles, respectively, at the primary analysis; and 1–38 and 1–42 cycles, respectively, at the final analysis), and a higher percentage of patients in the P+LDAC arm received >6 cycles of treatment (15.7% versus 8.2% for V+LDAC in the primary analysis and 22.5% versus 16.2% in the final analysis).

### Objective response

The primary analysis failed to show a statistically significant benefit of V+LDAC compared with P+LDAC in the primary endpoint; ORR was 25.2% in patients who received V+LDAC versus 16.8% in patients who received P+LDAC (OR 1.66 [95% CI, 0.95–2.89]; *P* = 0.071; Table 2). In the final analysis, the proportion of patients in the V+LDAC arm who achieved ORR was higher than in the P+LDAC arm (27.7% versus 17.1%; OR 1.88 [95% CI, 1.24–2.83]; *P* = 0.002; Table 2).

**Table 2:** Response Rates by Treatment Arm: Primary and Final Analyses

	Primary Analysis		Final Analysis	
	P+LDAC (n = 125)	V+LDAC (n = 246)	P+LDAC (n = 222)	V+LDAC (n = 444)
Patients who achieved CR, n (%)	12 (9.6)	23 (9.3)	27 (12.2)	67 (15.1)
Patients who achieved CRi, n (%)	9 (7.2)	39 (15.9)	11 (5.0)	56 (12.6)
Patients who achieved CR or CRi, n (%)	21 (16.8)	62 (25.2)	38 (17.1)	123 (27.7)
95% CI^*a*^	11.26–24.32	20.19–30.98	12.73–22.62	23.74–32.04
OR V+LDAC vs P+LDAC^*b*^	1.66		1.88	
95% CI	0.95–2.89		1.24–2.83	
*P*	0.071		0.002	
No response assessment/not evaluable for response, n (%)	16 (12.8)	95 (38.6)	39 (17.6)	158 (35.6)
Death ≤28 d after randomization, n (%)	4 (3.2)	27 (11.0)	8 (3.6)	52 (11.7)
Death >28 and ≤56 d after randomization, n (%)	7 (5.6)	30 (12.2)	16 (7.2)	50 (11.3)
Death >56 and ≤84 d after randomization, n (%)	0	8 (3.3)	2 (0.9)	18 (4.1)
Median OS, mo (95% CI)	6.5 (5.1–8.1)	4.8 (3.8–6.4)	6.5 (4.9–8.0)	5.6 (4.5–6.8)
HR V+LDAC vs P+LDAC	1.26		0.97	
95% CI	(0.9–1.7)		(0.8–1.2)	
*P*	0.11		0.76	
Median EFS, mo (95% CI)	3.1 (2.1–5.8)	2.8 (2.3–3.8)	2.8 (2.1–4.9)	3.3 (2.6–4.2)
HR V+LDAC vs P+LDAC	1.18		0.96	
95% CI	(0.9, 1.6)		(0.8, 1.2)	
*P*	0.26		0.67	
Median RFS, mo (95% CI)	NE (3.7–NE)	4.9 (3.6–13.4)	18.7 (11.3–NE)	13.1 (6.2–NE)
HR V+LDAC vs P+LDAC	1.26		1.37	
95% CI	(0.4–4.1)		(0.7–2.7)	

^*a*^Wilson’s CI.

^*b*^Odds ratio derived from a Cochran–Mantel–Haenszel test stratified by baseline ECOG PS and type of AML. OR > 1 favors V+LDAC.

CI = confidence interval; CR = complete remission; CRi = complete remission with incomplete blood count recovery; EFS = event-free survival; HR = hazard ratio; NE = nonevaluable; OR = odds ratio; OS = overall survival; P+LDAC = placebo plus low-dose cytarabine; RFS = relapse-free survival; V+LDAC = volasertib plus low-dose cytarabine.

The proportion of patients who had no response assessment or were not evaluable for response was higher in the V+LDAC arm compared with P+LDAC (38.6% versus 12.8% in the primary analysis and 35.6% versus 17.6% in the final analysis). The majority of these cases were due to early death prior to the planned first response assessment at the end of treatment cycle 2 (Table 2). These patients were included in the primary efficacy analysis, although no response data were available.

Subgroup analysis of ORR showed differences in response rates by gender, age, weight, ECOG PS, 2010 ELN genetic risk group,^13^ type of AML, *NPM1* mutation status, and geographical region of enrollment, with a trend toward better ORR with V+LDAC compared with P+LDAC in most subgroups (Table 3). Notably, in the ECOG 2 subgroup, the addition of volasertib to LDAC seemed to negatively impact on the outcome, whereas in the ECOG 0 and 1 subgroups, respectively, the response analyses indicate a potential benefit with the addition of volasertib. Subgroup analysis of other genetic aberrations found in AML, such as mutations in *FLT3* and *CEBPA*, was not conducted due to the small number of patients with these mutations in this trial.

**Table 3:**
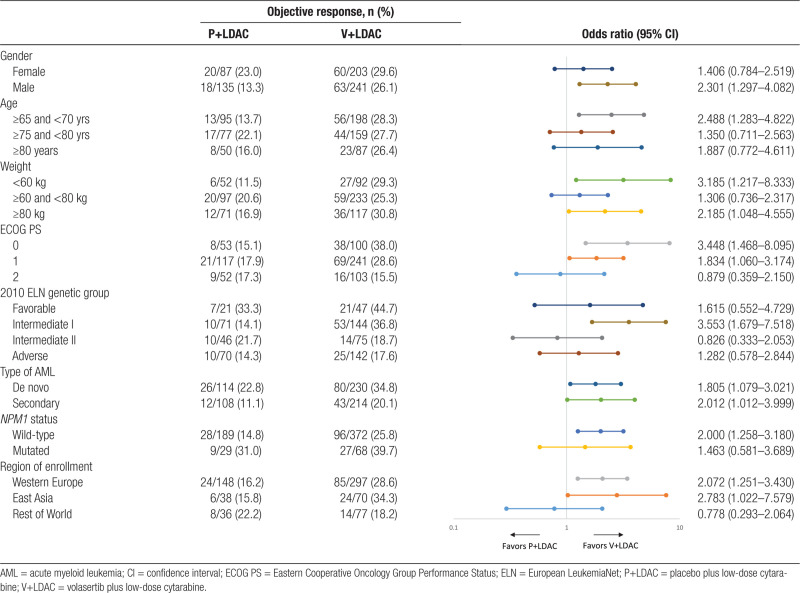
Objective Response Rate by Treatment Arm and in Various Subgroups: Final Analysis

### Overall survival

**Figure 2.**
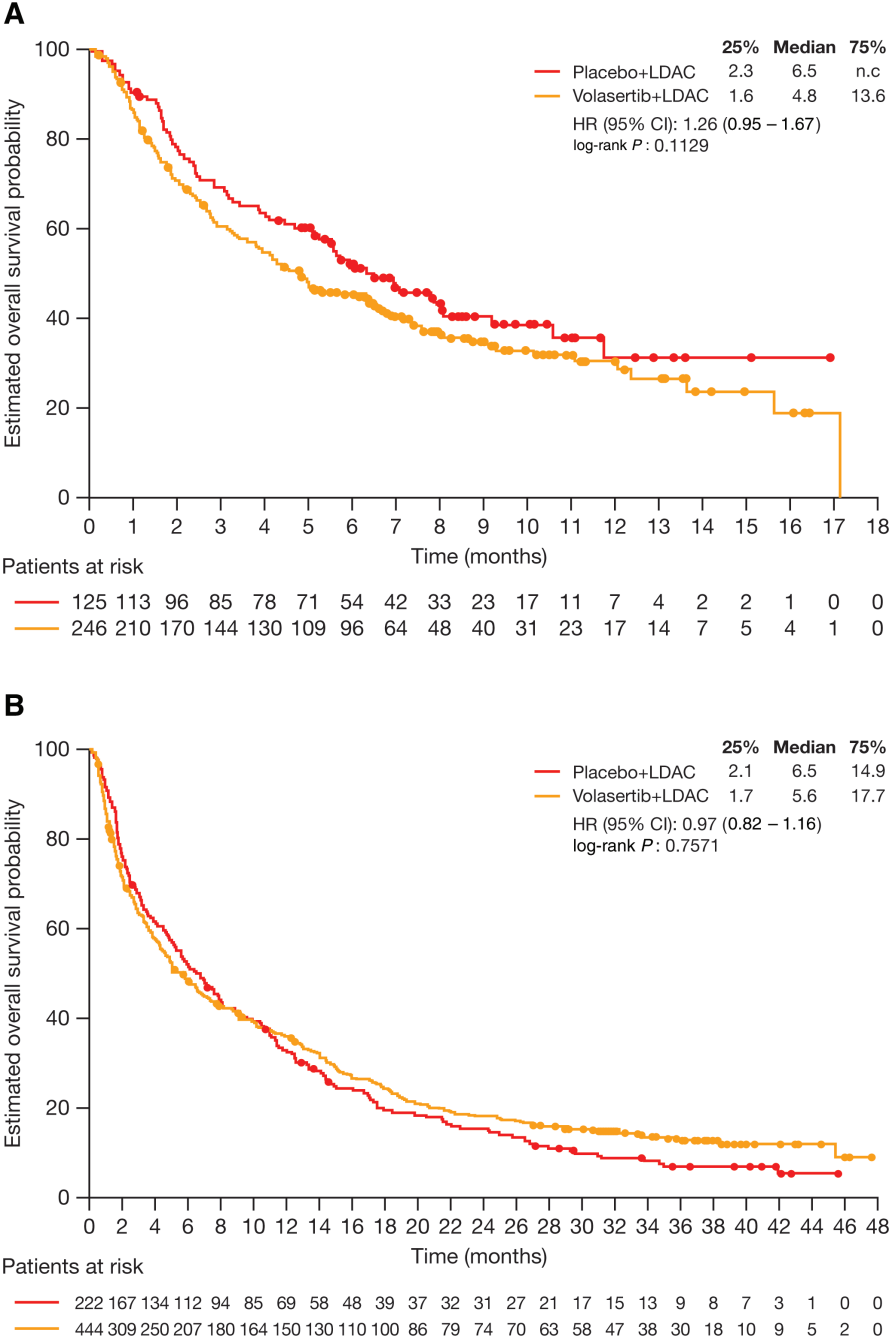
**Kaplan−Meier analysis of overall survival by treatment arm: primary analysis (A) and final analysis (B).** CI = confidence interval; HR = hazard ratio; LDAC = low-dose cytarabine; n.c. = not calculable.

In the primary analysis, numerically shorter, but not statistically significant, OS was seen for the V+LDAC arm compared with the P+LDAC arm (median 4.8 versus 6.5 months; HR 1.26 [95% CI, 0.95–1.67]; *P* = 0.113; Figure 2). At the final analysis, survival probability over time was similar between the 2 treatment arms, with a median OS of 5.6 months on V+LDAC and 6.5 months on P+LDAC (HR 0.97 [95% CI, 0.82–1.16]; *P* = 0.757; Figure 2).

Subgroup analyses of OS based on baseline factors are provided in Supplemental Table 1, Figures 1 and 2, http://links.lww.com/HS/A177. Of note, in the respective ECOG 0 and 1 subgroups, the addition of volasertib to LDAC appeared to indicate a potential benefit. In the ECOG 2 subgroup, however, the addition of volasertib appeared to negatively impact OS.

### Safety

Almost all patients experienced an on-treatment AE prior to final data cut-off (V+LDAC, 99.5%; P+LDAC, 97.7%; Supplemental Tables 2 and 3, http://links.lww.com/HS/A177). Across both arms, the most common AEs were infections/infestations (grouped term; V+LDAC, 81.3%; P+LDAC, 63.5%) and febrile neutropenia (V+LDAC, 60.4%; P+LDAC, 29.3%). The most commonly reported AEs in the individual arms were febrile neutropenia, thrombocytopenia, anemia, and neutropenia in the V+LDAC arm, and were nausea and pyrexia in the P+LDAC arm. The incidence of infections and infestations, and blood cytopenias were numerically higher in the V+LDAC arm than in the P+LDAC arm; Supplemental Table 3, http://links.lww.com/HS/A177). Patients in the V+LDAC arm had a higher incidence of grade ≥3 infections/infestations than patients in the P+LDAC arm (58.1% versus 38.3%; HR 1.77 [95% CI, 1.39–2.27]; *P* < 0.0001). Similarly, the incidence of febrile neutropenia was higher in patients receiving V+LDAC than in patients receiving P+LDAC (60.4% versus 29.3%; HR 2.84 [95% CI, 2.16–3.73]; *P* < 0.0001).

The incidence of grade ≥4 AEs was higher in the V+LDAC arm, compared with the P+LDAC arm. The most common grade 4 AEs in both arms were thrombocytopenia and neutropenia, and the difference in grade 4 AE frequency between treatment arms was driven by increased incidences of sepsis, febrile neutropenia, thrombocytopenia, anemia, neutropenia, and leukopenia in the V+LDAC arm. Importantly, AEs leading to death (grade 5) were reported with a higher frequency in the V+LDAC arm (31.2%) than in the P+LDAC arm (18.0%), potentially driven by a higher incidence of infections and infestations (17.1% versus 6.3%; Supplemental Table 3 and Figure 3, http://links.lww.com/HS/A177).

To further explore the difference in infectious complications between the treatment arms, we investigated the incidence, severity, and duration of neutropenia. Grades of neutropenia were similar between the treatment arms at baseline, with the lowest (grade 4) neutrophil levels reported in 39.1% and 46.4% of patients receiving P+LDAC and V+LDAC, respectively. However, over the course of treatment, more patients in the V+LDAC arm than in the P+LDAC arm experienced worsening of neutropenia, with grade 4 neutrophil values reported in 94.3% of patients receiving V+LDAC compared with 75.8% of patients receiving P+LDAC.

AEs in the grouped term mucositis were mostly of grade 1 or 2, but these AEs may have contributed to infectious complications. The frequency of any-grade mucositis (grouped term) was higher in patients receiving V+LDAC (33.3%) than in those receiving P+LDAC (12.6%). This difference in incidence between arms was driven by the most common AE terms in the grouped category, stomatitis and mucosal inflammation.

The majority of patients received treatment with antibiotics or antifungals during the study, and treatment with these was more frequent in the V+LDAC arm (antibiotics 95.2% and antifungals 76.1%) than in the P+LDAC arm (antibiotics 85.6% and antifungals 56.3%). The mean duration of antibiotic or antifungal use was similar between treatment arms.

### Exploratory analyses

Subsequent to the primary analysis, ad hoc exploratory analyses were conducted to understand the difference in outcomes between the previous phase 2 study^10^ and the current phase 3 trial, and the possible reasons why this phase 3 trial did not meet its primary endpoint. One possible cause is differences in cycle 1 dose intensities; protocols for the phase 2 study and this phase 3 trial had similar rules to allow doses to be delayed or skipped if required, resulting in decreased dose intensities. In the majority of patients in the current study, lower dose intensities were caused by a delayed start of the subsequent treatment cycle, that is, length of treatment cycle >28 days. In the phase 2 trial, patients received a lower median dose intensity of volasertib (17.6 mg/d) than in this phase 3 trial (20.8 mg/d). Patients receiving lower V+LDAC dose intensities in this phase 3 trial had longer OS, longer time to fatal AEs and fatal infections, and a higher ORR than did patients receiving V+LDAC at a higher dose intensity (Supplemental Figures 3–5 and Table 4, http://links.lww.com/HS/A177).

To determine whether use of prophylactic antibiotics affected the incidence of fatal infections, an analysis of the time to fatal infection by extent of prophylactic antibiotic treatment was conducted. Patients in the V+LDAC arm who were not treated with prophylactic antibiotics had a higher risk of fatal infections than patients who received any prophylactic antibiotics (Supplemental Figure 6, http://links.lww.com/HS/A177).

A competing risk analysis was performed to explore separately the effect of volasertib on OS events resulting from lack of efficacy or nontolerability. A benefit was observed in the V+LDAC arm compared with the P+LDAC arm when AML-related deaths were considered by the investigator as potentially due to lack of efficacy, whilst a benefit was observed in the opposite direction for deaths considered by the investigator as potentially due to intolerability (Supplemental Figures 7 and 8, http://links.lww.com/HS/A177).

## Discussion

The current randomized, double-blind, placebo-controlled, phase 3 trial was conducted to evaluate the efficacy and safety of volasertib, a highly potent and selective Plk inhibitor, combined with LDAC in previously untreated older patients with AML who were considered unsuitable for intensive chemotherapy, and aimed to confirm the encouraging results from the previous, randomized, open-label, phase 2 trial.^10^

The primary endpoint was not met; in the primary analysis, V+LDAC was not associated with significantly higher ORR compared with P+LDAC. In the final analysis, the proportion of patients who achieved an objective response was higher in the V+LDAC arm than in the P+LDAC arm; however, a substantially greater number of patients receiving V+LDAC had no response assessment or were not evaluable, primarily because of a higher death rate prior to the first response assessment at the end of cycle 2. In the subgroup analysis, the addition of volasertib to LDAC in patients in the ECOG 2 subgroup seemed to negatively impact the ORR.

In the primary analysis, the numerically shorter OS observed in the V+LDAC arm, in comparison with the P+LDAC arm, was likely due to a higher frequency of fatal infections in patients receiving V+LDAC. The study was subsequently unblinded, which may have influenced subsequent patient management, medical decision making, and, consequently, the outcomes seen in the trial. The final analysis, which demonstrated no difference in OS between treatment arms should, therefore, be considered exploratory and descriptive only. The competing risk modeling of survival endpoints indicated fewer deaths potentially due to lack of efficacy, but more deaths potentially due to intolerability, in the V+LDAC arm compared with P+LDAC arm. Such competing risk analyses are particularly important for oncology studies of elderly patients, since many older patients may die of non-cancer-related causes rather than from a lack of treatment efficacy.^14^ The particularly adverse OS of patients with ECOG PS 2 treated with V+LDAC contributed to the OS trend in the V+LDAC arm, most likely because frailer patients were at higher risk of severe AEs associated with volasertib treatment. Although previous studies have reported very low rates or absence of remissions with LDAC for patients with an adverse genetic profile,^4,5^ in the final analysis of this study, CR or CRi was reported in 14.3% of patients in the adverse genetic group who received P+LDAC, and in 17.6% of patients who received V+LDAC.

More grade ≥4 AEs and almost twice as many grade 5 AEs were reported in the V+LDAC arm compared with the P+LDAC arm. This was attributed to the more pronounced myelosuppression observed in the V+LDAC treatment group, in addition to the higher reported frequency of mucositis. These results were expected based on the mode of action of volasertib and on previous clinical studies;^15–18^ volasertib was expected to transiently inhibit the proliferation of normal dividing cells, leading to temporary myelosuppression, and increasing the risk of associated complications such as febrile neutropenia, infections, or thrombocytopenic bleeding.

The results of our exploratory analyses suggested that differences in dose intensity may have influenced outcomes. Median dose intensities resulted from medical assessment and decision-making by investigators; dose intensities were different between the previous phase 2 study and the current phase 3 study, although both studies had similar rules to adapt dosing. Additionally, the open-label nature of the phase 2 study versus the double-blind phase 3 design might have influenced medical assessment and decision making and, thus, dose intensity. Patients receiving a lower dose intensity of volasertib (and therefore also LDAC) in this phase 3 trial had a longer time to fatal AEs and fatal infections, which were some of the major factors contributing to the poorer OS in the V+LDAC arm compared to the P+LDAC arm.

Supportive care could potentially influence outcomes, and improvement of supportive care with the compulsory administration of prophylactic antibiotics/antifungals and blood transfusions may be advisable to proactively manage treatment-induced myelosuppression and avoid infections. Of note, the recommendations for supportive care were similar across the phase 2 and phase 3 studies, both of which allowed supportive care use at the investigator’s discretion. The results of our exploratory analyses suggest that prophylactic antibiotics may reduce the risk of fatal infections in patients treated with volasertib. Effective supportive care, along with reduction in dose intensity, may improve tolerability in patients receiving volasertib combination therapy, and ultimately improve OS.

A numerically higher ORR but no corresponding increase in survival for V+LDAC compared with P+LDAC is consistent with the results of trials testing other novel agents in older patients with AML who are ineligible for intensive chemotherapy. Clofarabine showed significantly superior ORRs compared with LDAC, but failed to show a survival benefit because the increased remission rate was obtained at a cost of greater toxicity.^19^ Addition of gemtuzumab ozogamicin to LDAC improved ORR but did not improve OS due to inferior survival after relapse. Additionally, in patients who did not achieve remission, survival was inferior in those who received the combination in comparison to those who received LDAC alone.^20^

As a result of the observed disparity between response rate and survival outcomes, there is ongoing debate as to whether the response rate is a good predictor of OS and whether it is suitable as a surrogate endpoint in trials of AML.^21,22^ A meta-analysis of 20 trials in AML showed a significant correlation between rates of CRi or better and median OS,^23^ supporting the use of CR plus CRi as the primary endpoint in this study.

At the time this study was designed, LDAC was considered the standard treatment for patients with AML who were ineligible for standard intensive chemotherapy. Since then, the hypomethylating agents azacitidine and decitabine have been introduced into therapy guidelines as recommended treatment for these patients.^5^ These agents may now be considered the preferred combination partners and comparators for clinical trials. Furthermore, in the phase 1b/2 M14-358 and phase 1b M14-387 studies, the BCL-2 inhibitor, venetoclax, in combination with azacitidine, decitabine, or LDAC, demonstrated encouraging CR rates and remission duration in AML patients of older age (≥60 yrs) or with comorbidities precluding the use of intensive induction chemotherapy. The pivotal phase 3 VIALE-A trial reported that, in patients with AML who were ineligible for intensive induction therapy due to comorbidities or age, treatment with venetoclax and azacitidine led to a significant improvement in OS (14.7 versus 9.6 mo, *P* < 0.001), composite complete remission (CR + CRi; 66.4% versus 28.3%, *P* < 0.001) and event-free survival (9.8 versus 7.0 months, *P* < 0.001), compared to treatment with placebo and azacytidine.^24^ Venetoclax in combination with a hypomethylating agent or LDAC therefore offer new therapy options for these patients.^25–27^

This randomized phase 3 trial did not meet its primary endpoint of ORR in the primary analysis, and did not confirm the survival benefits of volasertib in combination with LDAC seen in a previous randomized phase 2 study.^10^ There was a notably higher rate of fatal infections in patients who received V+LDAC, indicating that the volasertib dose and schedule used were not sufficiently tolerable. Development of volasertib was discontinued in 2018, following a strategic decision by the sponsor. Nevertheless, the results of this trial provide insight into the efficacy and tolerability of volasertib in older patients with AML, and may inform development of other Plk1 inhibitors.

## Acknowledgments

The authors thank all participating patients and their families, the medical and nursing staff, and the other Study 1230.14 investigators for their contribution to this study. A complete list of investigators is provided in the Supplemental Digital Material, http://links.lww.com/HS/A177. Medical writing support for the development of this manuscript, under the direction of the authors, was provided by Hannah Simmons, MSc, of Ashfield MedComms, an Ashfield Health company. 

## Disclosures

HD reports receipt of consultancy fees from AbbVie, Agios, Amgen, Astellas Pharma, Astex Pharmaceuticals, AstraZeneca, Bristol-Myers Squibb, Celgene, GEMoaB, Helsinn, Janssen, Jazz Pharmaceuticals, Novartis, Oxford BioMedica, and Roche; and receipt of grants or funds from Agios, Amgen, Astellas, Bristol-Myers Squibb, Celgene, Jazz Pharmaceuticals, Novartis, and Pfizer. AS reports honoraria directly received from AbbVie, Amgen, Bristol-Myers Squibb, Gilead, Janssen, Novartis, Pfizer, Roche, Sanofi-Genzyme, and Takeda; receipt of consultancy fees from AbbVie, Bristol-Myers Squibb, Gilead, Janssen, Novartis, Pfizer, Roche, Sanofi-Genzyme, and Takeda; and receipt of grants or funds from AbbVie, Alexion, Amgen, Astellas, Bristol-Myers Squibb, DEMO Pharmaceuticals, Genesis, Gilead, Janssen, Merck Sharp & Dohme, Novartis, Pfizer, raPHARM, Roche, Sanofi-Genzyme, Takeda, and WinMedica. WF reports participation on advisory boards for Morphosys, AbbVie, Pfizer, Amgen, Jazz Pharmaceuticals; support for meeting attendance from Amgen, Jazz Pharmaceuticals, Daiichi Sankyo Oncology, Bristol-Myers Squibb, and Servier; and receipt of support in medical writing from Amgen, Boehringer Ingelheim, Pfizer, and AbbVie. H-JK reports receipt of consultancy fees from Amgen, AML Global Portal, Astellas Pharma, Celgene, Novartis, SL VaxiGen, Yuhan Pharmaceuticals, Eutilex, and Otsuka Pharmaceuticals; and royalties of payments for lectures/manuscript preparations from Astella, AML Global Portal, Novartis, and AbbVie. J-HL reports receipt of grants or funds from Boehringer Ingelheim. KU reports honoraria directly received from Novartis, Astellas Pharma, Alexion, Eisai, Merck Sharp & Dohme, Otuska, Ono, Kyowa-Kirin, SymBio, Celgene, Daiichi Sankyo, Takeda, Nihon Shinyaku, PharmaEssentia, and Bristol-Myers Squibb; receipt of grants or funds from Astellas Pharma, AbbVie, Apelis, Otsuka Pharmaceutical, Daiichi Sankyo, Novartis, Merck Sharp & Dohme, Chugai, Pfizer, Bristol-Myers Squibb, Alexion, Ono, Janssen, SymBio, Celgene, Daiichi Sankyo, Sumitomo Dainippon, Takeda, Nihon Shinyaku, Novartis, Boehringer Ingelheim, Mundipharma, Chugai Pharma, Gilead, and Kyowa Kirin; receipt of grants/pending grants from Astellas Pharma, Otsuka Pharmaceutical, Apelis, SymBio, Takeda, Nihon Shinyaku, Novartis, Merck Sharp & Dohme, Alexion, Kyowa Kirin, Gilead, Chugai, Pfizer, Bristol-Myers Squibb, and Daiichi Sankyo; and receipt of royalties of payments for lectures/manuscript preparations from Novartis, Astellas Pharma, Alexion, Eisai, Merck Sharp & Dohme, Otuska, Ono, Kyowa-Kirin, Celgene, Daiichi Sankyo, Takeda, Nihon Shinyaku, PharmaEsseitia, Bristol-Myers Squibb, and Yakurt. H-AH reports receipt of consultancy fees from Amgen. CR reports receipt of grants/pending grants from AbbVie, Amgen, Novartis, Celgene, Jazz Pharmaceuticals, Agios, Daiichi Sankyo, Astellas Pharma, Sunesis, Roche, and MaaT Pharma. PR reports receipt of research grants from Incyte and Pfizer. IS reports honoraria received directly from Celgene, Bristol-Myers Squibb, Pfizer, Takeda, Janssen, and Amgen. FT reports participation on advisory boards for Celgene, Novartis, Jazz Pharmaceuticals, AbbVie, Astellas Pharma, and Pfizer. DJD reports receipt of consultancy fees from Amgen, Autolus, Agios, Blueprint Medicines, Forty-Seven, Incyte, Jazz Pharmaceuticals, Novartis, Pfizer, Servier, and Takeda; and research funding from Abbvie, GlycoMimetics, Novartis, and Blueprint Pharmaceuticals. MS reports participation on an advisory council or committee for Bristol-Myers Squibb, Celgene, Takeda, Millenium, and Pfizer. VB is an employee of SCS Boehringer Ingelheim Comm.V. MG is an employee of Boehringer Ingelheim Pharm. TT is an employee of Boehringer Ingelheim International GmbH. All the other authors have no conflicts of interest to disclose.

## Sources of funding

This study was funded by Boehringer Ingelheim.

## Study group members

Anagnostopoulos, Achilleas

Anagnostopoulos, Nikolaos

Ando, Kiyoshi

Aulitzky, Walter

Baltasar, Patricia

Bergeron, Julie

Bernard, Marc

Borbenyi, Zita

Briasoulis, Evangelos

Bulabois, Claude Eric

Cairoli, Roberto

Calbecka, Malgorzata

Capra, Marcelo

Chang, Cheng-Shyong

Choudhury, Ratnamala

Chromik, Jorg

Cortes, Jorge

Coutinho, Jorge

De Prijck, Bernard

Deeren, Dries

Delaunay, Jacques

Demeter, Judit

Döhner, Hartmut

Esteve, Jordi

Esteves, Graca

Fiedler, Walter

Fujisawa, Shin

Gasztonyi, Zoltan

Geissler, Klaus

Gjertsen, Bjorn Tore

Goetze, Katharina

Gomez, David

Goueli, Basem

Graux, Carlos

Guimaraes, Jose

Havelange, Violaine

Hogge, Donna

Horst, Heinz-August

Hou, Hsin-An

Iida, Hiroatsu

Jindra, Pavel

Jung, Chul Won

Kameoka, Yoshihiro

Kaporskaya, Tatiana

Kim, Hee-Je

Kim, Hyeoung Joon

Kindler, Thomas

Kobayashi, Yukio

Koh, Youngil

Kohler, Friedemann

Kraemer, Alwin

Krause, Stefan

Krauter, Juergen

Lee, Je Hwan

Maeda, Yoshinobu

Maertens, Johan

Mariz, Mario

Marmont, Filippo

Marolleau, Jean-Pierre

Martínez, Pilar

McDonald, Andrew

Minami, Hironobu

Miyamoto, Toshihiro

Miyazaki, Yasushi

Mohty, Mohamad

Mueller-Tidow, Carsten

Nakamae, Hirohisa

Neubauer, Andreas

Noppeney, Richard

Novak, Jan

Olga, Salamero

Onishi, Yasushi

Ossenkoppele, Gert

Pichler, Angelika

Pigneux, Arnaud

Pires, Andiara

Porkka, Kimmo

Recher, Christian

Rego, Eduardo

Reichle, Albrecht

Reman, Oumedaly

Riscala, Carlos

Robak, Tadeusz

Rossi, Giuseppe

Rousselot, Philippe

Salmenniemi, Urpu

Samoylova, Olga

Sandhu, Irwindeep

Sanz, Miguel Angel

Schiller, Gary

Schmid, Christoph

Selleslag, Dominik

Shneider, Tatiana

Sierra, Jorge

Sill, Heinz

Storring, John

Straetmans, Nicole

Strickland, Stephen

Susana, Vives

Symeonidis, Argiris

Theunissen, Koen

Thol, Felicitas

Thomas, Xavier

Turlure, Pascal

Uchida, Toshiki

Ueda, Yasunori

Usuki, Kensuke

Vey, Norbert

Vidriales, Belén

Waleed, Ghanima

Westermann, Joerg

Wiebe, Stefanie

Wulf, Gerald

Yamauchi, Takahiro

Yee, Karen

Zak, Pavel

## Supplementary Material


