# Supplementary material for: Adjunctive Volasertib in Patients With Acute Myeloid Leukemia not Eligible for Standard Induction Therapy: A Randomized, Phase 3 Trial
Source: Hemasphere. 2021 Aug 2;5(8):e617. doi: 10.1097/HS9.0000000000000617 (PMC8328241; doi:10.1097/HS9.0000000000000617)
Supplement: Supplementary file 1 [file hs9-5-e617-s001.docx]

**Supplemental Digital Content (SDC) for Döhner et al. Adjunctive Volasertib in Patients With Acute Myeloid Leukemia not Eligible for Standard Induction Therapy: A Randomized, Phase 3 Trial**

**SDC Methods. Unplanned, exploratory, post hoc analysis to compare the phase 2 and phase 3 results**

Objective response rate (ORR), overall survival (OS), time to fatal adverse events (AEs), and time to fatal infections were analyzed retrospectively by dose intensity. Dose intensity was calculated by dividing the dose of volasertib or placebo administered in Cycle 1 by the interval (days) from start of Cycle 1 to start of Cycle 2 (with the dose intensity per protocol [2 x 350 mg in a 28-day cycle] being 25 mg/d). Higher and lower dose intensities were defined as a calculated dose of either volasertib or placebo of ≥25 mg/d and <25 mg/d, respectively, with dose intensities <25 mg/d resulting from skipped doses or delays in the start of the next cycle; of note, skipped or delayed doses will also have reduced the dose intensity of LDAC in parallel. Differences in dose intensities were the result of medical assessment and decision making by the investigator, and imbalances in patient and/or disease characteristics between the dose intensity groups were expected. Therefore, to minimize the confounding effect from baseline imbalance, propensity score methodology was used to identify a subset of patients with similar baseline factors between the dose intensity groups. Competing risk modeling was conducted to compare treatment effect and determine to what extent the OS results observed in this trial were caused by the intolerability or lack of efficacy of volasertib. Several approaches were taken to identify the OS events caused by lack of efficacy, treatment intolerability, or neither of the two. Survival analysis with competing risks was conducted using the categorized data based on two standard models: the cause-specific hazard model and the subdistribution model.^1,2^

**SDC References**

1. Prentice RL, Kalbfleisch JD, Peterson AV, Jr., Flournoy N, Farewell VT, Breslow NE. The analysis of failure times in the presence of competing risks. *Biometrics*. 1978;34:541–554.

2. Fine JP, Gray RJ. A proportional hazards model for the subdistribution of a competing risk. *J Am Stat Assoc*. 1999;94:496–509.

| **SDC Table 1**  **Overall Survival Rate by Treatment Arm and in Various Subgroups: Final Analysis.** | | | | |
| --- | --- | --- | --- | --- |
|  | **Median OS, months (95% CI)** | | **Hazard ratio (95% CI)** | |
|  | **P+LDAC** | **V+LDAC** |  |  |
| BSA (m^2^) |  |  |  |  |
| <1.6 | 10.5 (5.9– 14.3) | 6.1 (3.4–10.1) |  | 1.157 (0.768–1.742) |
| ≥1.6 and <1.8 | 8.0 (3.5–12.5) | 5.0 (3.6–7.8) |  | 1.026 (0.760–1.385) |
| ≥1.8 | 5.1 (3.1–6.7) | 5.7 (4.3–8.2) |  | 0.818 (0.638–1.050) |
| Age (years) |  |  |  |  |
| ≥65 and <75 | 4.8 (3.4–6.9) | 6.5 (4.5–8.6) |  | 0.822 (0.633–1.066) |
| ≥75 and <80 | 7.6 (4.6–11.0) | 4.9 (3.3–6.8) |  | 1.023 (0.766–1.366) |
| ≥80 | 8.2 (5.6–12.3) | 5.3 (2.0–10.1) |  | 1.050 (0.718–1.534) |
| Weight (kg) |  |  |  |  |
| <60 | 9.3 (5.9–12.5) | 6.1 (3.9–11.3) |  | 0.996 (0.686–1.446) |
| ≥60 and <80 | 6.7 (3.5–10.6) | 4.6 (3.4–6.5) |  | 0.976 (0.760–1.254) |
| ≥80 | 5.6 (2.8–7.1) | 6.6 (4.6–9.8) |  | 0.832 (0.608–1.138) |
| ECOG PS |  |  |  |  |
| 0 | 9.2 (6.0–11.3) | 11.7 (6.5–15.2) |  | 0.810 (0.562–1.168) |
| 1 | 5.6 (3.5–7.9) | 6.4 (4.9–8.6) |  | 0.828 (0.654–1.049) |
| 2 | 5.6 (2.2–12.3) | 2.0 (1.6–2.8) |  | 1.475 (1.041–2.091) |
|  |  |  |  |  |
|  |  |  |  |  |
| 2010 ELN genetic group |  |  |  |  |
| Favorable | 11.3 (5.7–19.8) | 13.3 (6.1–28.9) |  | 0.693 (0.388–1.235) |
| Intermediate I | 6.7 (4.5–10.3) | 9.3 (4.5–12.4) |  | 0.823 (0.604–1.121) |
| Intermediate II | 10.9 (4.1–15.0) | 6.4 (4.0–9.1) |  | 1.227 (0.829–1.815) |
| Adverse | 4.9 (1.9–7.1) | 3.5 (2.5–4.8) |  | 1.067 (0.794–1.434) |
| Type of AML |  |  |  |  |
| De novo | 7.9 (5.3–12.3) | 7.2 (4.9–10.3) |  | 0.916 (0.716–1.172) |
| Secondary | 5.6 (3.1–7.6) | 4.8 (3.8–5.8) |  | 0.941 (0.740–1.196) |
| *NPM1* status |  |  |  |  |
| Wildtype | 6.0 (4.8–8.1) | 5.3 (4.3–6.8) |  | 0.941 (0.782–1.132) |
| Mutated | 7.0 (3.0–17.5) | 7.8 (2.9–13.9) |  | 0.845 (0.519–1.376) |
| Region of enrollment |  |  |  |  |
| Western Europe | 5.7 (4.1–7.8) | 5.1 (3.9–6.6) |  | 0.895 (0.726–1.102) |
| East Asia | 11.2 (5.5–14.3) | 11.2 (6.8–14.9) |  | 0.814 (0.528–1.254) |
| Rest of World | 6.7 (2.8–12.5) | 2.9 (1.7–4.6) |  | 1.253 (0.812–1.932) |
|  |  |  |  |  |

AML=acute myeloid leukemia, BSA=body surface area, CI=confidence interval, ECOG PS=Eastern Cooperative Oncology Group Performance Status, ELN=European LeukemiaNet, P+LDAC=placebo plus low-dose cytarabine, V+LDAC=volasertib plus low-dose cytarabine.

| **SDC Table 2**  **Summary of Adverse Events at Final Analysis.** | | |
| --- | --- | --- |
| **AE, n (%)** | **P+LDAC (n=222)** | **V+LDAC (n=439)** |
| Any AE | 217 (97.7) | 437 (99.5) |
| Drug-related AEs^a^ | 163 (73.4) | 352 (80.2) |
| AE leading to dose reduction^b^ | 16 (7.2) | 33 (7.5) |
| AEs leading to discontinuation of study drugs | 30 (13.5) | 74 (16.9) |
| Due to progressive disease | 12 (5.4) | 15 (3.4) |
| Due to other AE | 18 (8.1) | 59 (13.4) |
| Serious AEs | 163 (73.4) | 380 (86.6) |
| Fatal AEs | 40 (18.0) | 137 (31.2) |
| CTCAE grade ≥3 | 192 (86.5) | 419 (95.4) |

AE=adverse event, CTCAE=Common Terminology Criteria for Adverse Events, P+LDAC=placebo plus low-dose cytarabine, V+LDAC=volasertib plus low-dose cytarabine.

^a^As assessed by the investigator.

^b^All AEs leading to dose reduction are assumed to be AEs leading to a reduction of the volasertib/placebo dose.

| **SDC Table 3**  **Adverse Events Occurring in >10% of Patients at the Preferred Term Level in Patients in Either Treatment Arm: Final Analysis.** | | | | | | | | |
| --- | --- | --- | --- | --- | --- | --- | --- | --- |
| **AE, n (%)** | **P+LDAC (n=222)** | | | | **V+LDAC (n=439)** | | | |
|  | **All grades** | **Grade 3** | **Grade 4** | **Grade 5** | **All grades** | **Grade 3** | **Grade 4** | **Grade 5** |
| Any AE | 217 (97.7) | 63 (28.4) | 89 (40.1) | 40 (18.0) | 437 (99.5) | 84 (19.1) | 198 (45.1) | 137 (31.2) |
| Infections and infestations | 141 (63.5) | 51 (23.0) | 20 (9.0) | 14 (6.3) | 357 (81.3) | 136 (31.0) | 44 (10.0) | 75 (17.1) |
| Pneumonia | 44 (19.8) | 23 (10.4) | 8 (3.6) | 6 (2.7) | 124 (28.2) | 59 (13.4) | 18 (4.1) | 23 (5.2) |
| Sepsis | 10 (4.5) | 4 (1.8) | 1 (0.5) | 3 (1.4) | 51 (11.6) | 18 (4.1) | 14 (3.2) | 17 (3.9) |
| Blood and lymphatic system disorders | 138 (62.2) | 56 (25.2) | 73 (32.9) | 1 (0.5) | 351 (80.0) | 113 (25.7) | 225 (51.3) | 5 (1.1) |
| Febrile neutropenia | 65 (29.3) | 58 (26.1) | 4 (1.8) | 1 (0.5) | 265 (60.4) | 202 (46.0) | 51 (11.6) | 5 (1.1) |
| Thrombocytopenia | 66 (29.7) | 12 (5.4) | 53 (23.9) | - | 177 (40.3) | 14 (3.2) | 155 (35.3) | - |
| Anemia | 61 (27.5) | 41 (18.5) | 13 (5.9) | - | 148 (33.7) | 85 (19.4) | 44 (10.0) | - |
| Neutropenia | 36 (16.2) | 9 (4.1) | 27 (12.2) | - | 132 (30.1) | 9 (2.1) | 119 (27.1) | - |
| Leukopenia | 23 (10.4) | 8 (3.6) | 9 (4.1) | - | 44 (10.0) | 3 (0.7) | 40 (9.1) | - |
| Gastrointestinal disorders | 156 (70.3) | 25 (11.3) | 3 (1.4) | - | 330 (75.2) | 57 (13.0) | 4 (0.9) | 1 (0.2) |
| Nausea | 79 (35.6) | 3 (1.4) | - | - | 122 (27.8) | 4 (0.9) | - | - |
| Constipation | 55 (24.8) | 1 (0.5) | - | - | 121 (27.6) | 3 (0.7) | - | - |
| Diarrhea | 49 (22.1) | 4 (1.8) | - | - | 121 (27.6) | 8 (1.8) | - | - |
| Stomatitis | 16 (7.2) | 3 (1.4) | - | - | 80 (18.2) | 10 (2.3) | - | - |
| Vomiting | 27 (12.2) | - | - | - | 71 (16.2) | - | - | - |
| Abdominal pain | 26 (11.7) | - | - | - | 47 (10.7) | 2 (0.5) | - | - |
| General disorders and administration site conditions | 154 (69.4) | 25 (11.3) | 3 (1.4) | - | 302 (68.8) | 63 (14.4) | 17 (3.9) | 8 (1.8) |
| Pyrexia | 72 (32.4) | 3 (1.4) | 1 (0.5) | - | 102 (23.2) | 17 (3.9) | - | - |
| Peripheral edema | 42 (18.9) | 1 (0.5) | - | - | 76 (17.3) | 4 (0.9) | - | - |
| Asthenia | 44 (19.8) | 7 (3.2) | - | - | 69 (15.7) | 17 (3.9) | 2 (0.5) | - |
| Mucosal inflammation | 11 (5.0) | 1 (0.5) | - | - | 68 (15.5) | 7 (1.6) | 4 (0.9) | - |
| Fatigue | 33 (14.9) | 7 (3.2) | - | - | 64 (14.6) | 9 (2.1) | 1 (0.2) | - |
| Metabolism and nutrition disorders | 99 (44.6) | 15 (6.8) | 6 (2.7) | - | 229 (52.2) | 74 (16.9) | 15 (3.4) | 1 (0.2) |
| Hypokalemia | 38 (17.1) | 6 (2.7) | 3 (1.4) | - | 120 (27.3) | 37 (8.4) | 4 (0.9) | - |
| Decreased appetite | 45 (20.3) | 2 (0.9) | - | - | 82 (18.7) | 10 (2.3) | - | - |
| Respiratory, thoracic and mediastinal disorders | 103 (46.4) | 17 (7.7) | 5 (2.3) | 5 (2.3) | 234 (53.3) | 43 (9.8) | 19 (4.3) | 9 (2.1) |
| Cough | 21 (9.5) | - | - | - | 92 (21.0) | 4 (0.9) | - | - |
| Dyspnea | 32 (14.4) | 10 (4.5) | 1 (0.5) | - | 77 (17.5) | 17 (3.9) | 3 (0.7) | - |
| Epistaxis | 28 (12.6) | 1 (0.5) | - | - | 77 (17.5) | 4 (0.9) | 2 (0.5) | - |
| Skin and subcutaneous disorders | 93 (41.9) | 1 (0.5) | 1 (0.5) | - | 222 (50.6) | 27 (6.2) | 2 (0.5) | - |
| Petechiae | 24 (10.8) | - | - | - | 73 (16.6) | 4 (0.9) | - | - |
| Rash | 30 (13.5) | 1 (0.5) | - | - | 68 (15.5) | 8 (1.8) | - | - |
| Nervous system disorders | 65 (29.3) | 11 (5.0) | 1 (0.5) | 2 (0.9) | 144 (32.8) | 22 (5.0) | 5 (1.1) | 5 (1.1) |
| Headache | 23 (10.4) | - | - | - | 48 (10.9) | 1 (0.2) | - | - |
| Vascular disorders | 60 (27.0) | 8 (3.6) | - | - | 137 (31.2) | 23 (5.2) | 4 (0.9) | 1 (0.2) |
| Hematoma | 20 (9.0) | - | - | - | 56 (12.8) | 2 (0.5) | - | - |
| Musculoskeletal and connective tissue disorders | 81 (36.5) | 15 (6.8) | - | - | 150 (34.2) | 13 (3.0) | - | - |
| Back pain | 18 (8.1) | 1 (0.5) | - | - | 50 (11.4) | 3 (0.7) | - | - |

AE=adverse event, P+LDAC=placebo plus low-dose cytarabine, V+LDAC=volasertib plus low-dose cytarabine.

| **SDC Table 4**  **Objective Response by Treatment and Cycle 1 Dose Intensity – Primary Analysis.** | | | |
| --- | --- | --- | --- |
|  | **P+LDAC** | | **V+LDAC** |
| Lower dose intensity in Cycle 1  Patients randomized, n (%)  Objective response, n (%) | | 52 (100.0)  6 (11.5) | 117 (100.0)  48 (41.0) |
| Higher dose intensity in Cycle 1  Patients randomized, n (%)  Objective response, n (%) | | 41 (100.0)  15 (36.6) | 42 (100.0)  12 (28.6) |

P+LDAC=placebo plus low-dose cytarabine, V+LDAC=volasertib plus low-dose cytarabine.

**SDC Figure 1. Kaplan–Meier plots of overall survival by baseline ECOG PS in the final analysis.** CI=confidence interval, ECOG PS=Eastern Cooperative Oncology Group Performance Status, LDAC=low-dose cytarabine.





**
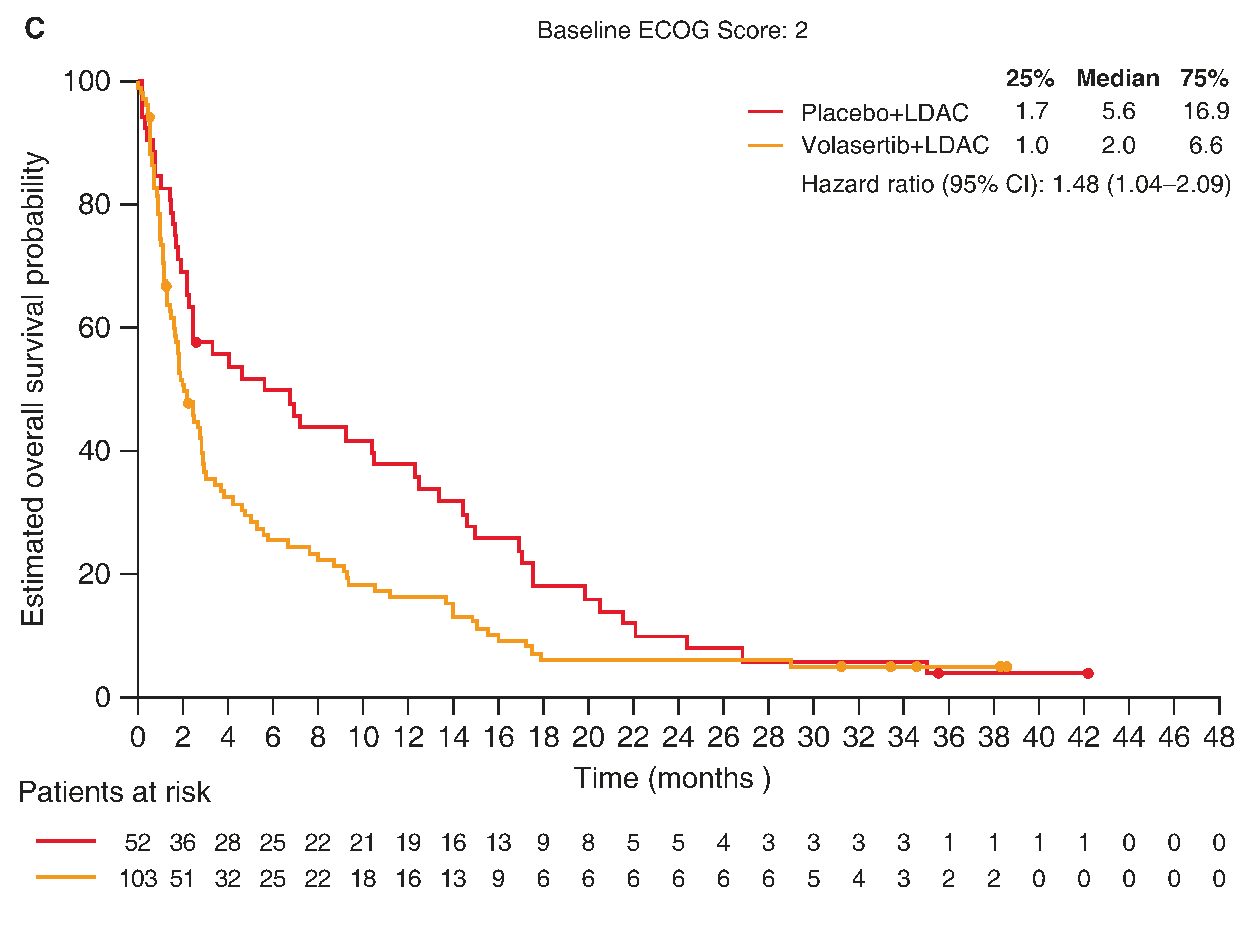
**

**SDC Figure 2. Kaplan–Meier plots of overall survival by geographical location in the final analysis.** CI=confidence interval, LDAC=low-dose cytarabine.





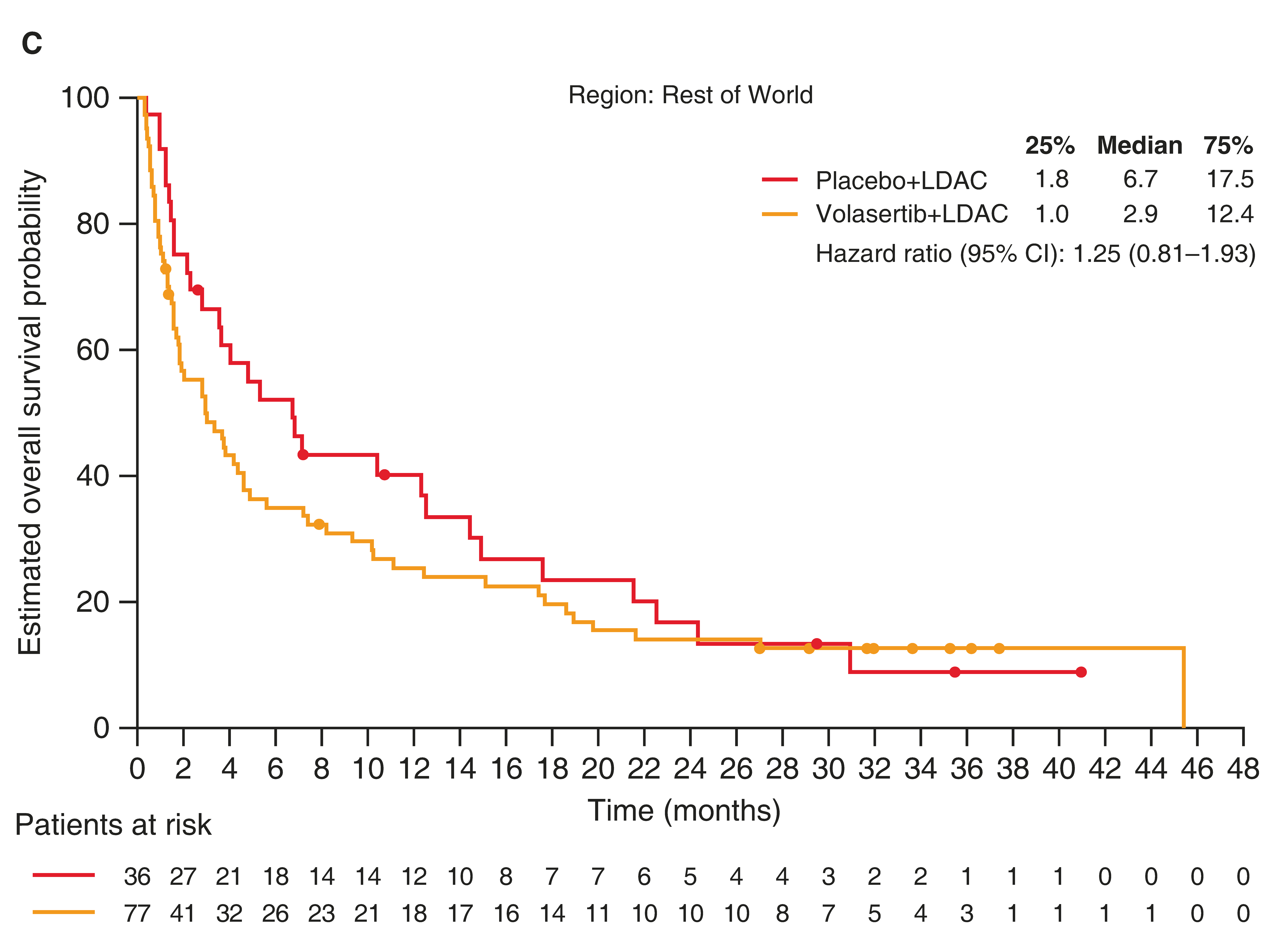


**SDC Figure 3. Kaplan–Meier plot of time to fatal infections by dose intensity of V+LDAC in the primary analysis set.** C1=cycle 1, DD=dose density, LDAC=low-dose cytarabine.

**
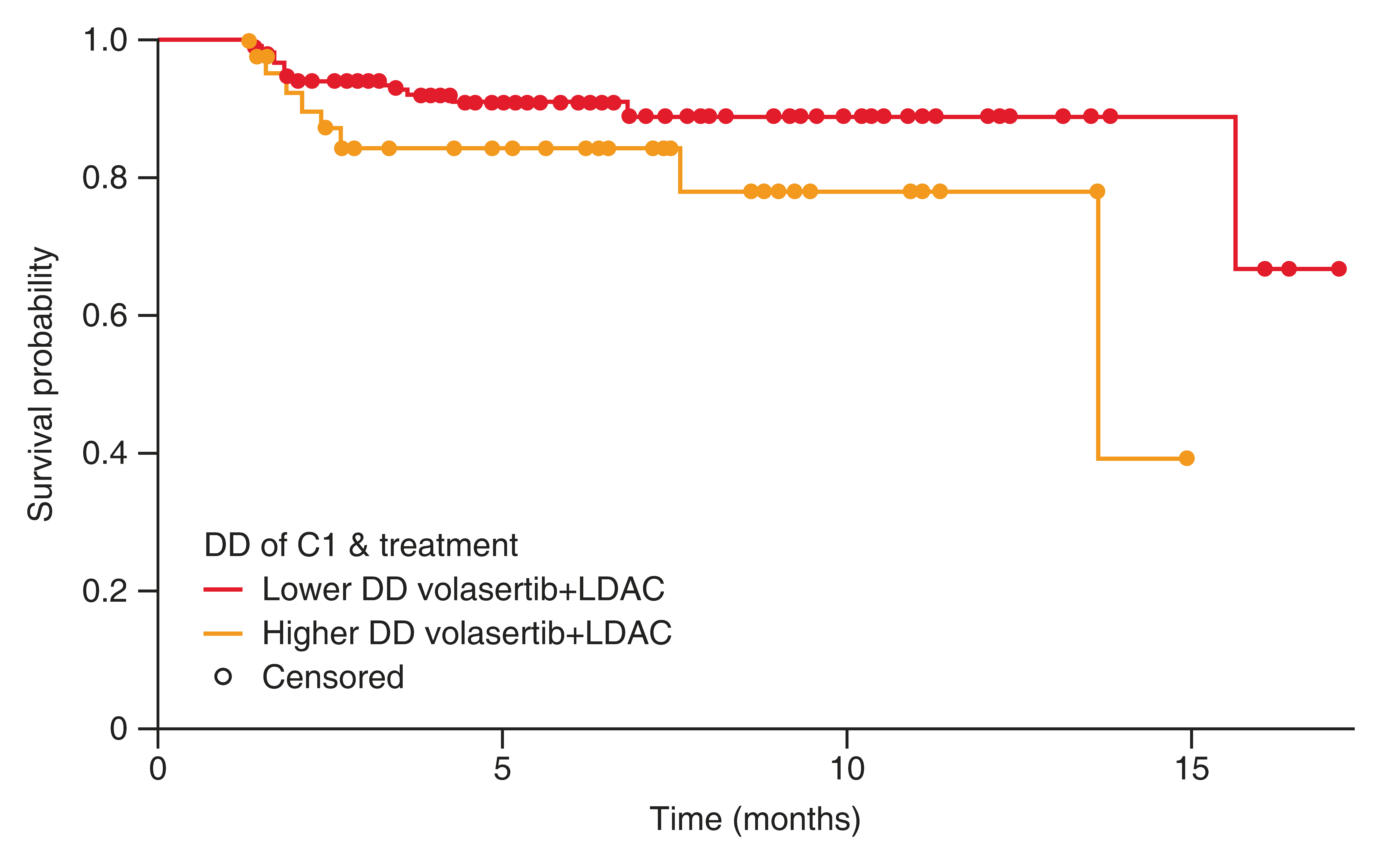
**

**SDC Figure 4. Kaplan–Meier plot of time to fatal adverse events by dose intensity of V+LDAC in the primary analysis.** C1=cycle 1, DD=dose density, LDAC=low-dose cytarabine.

**
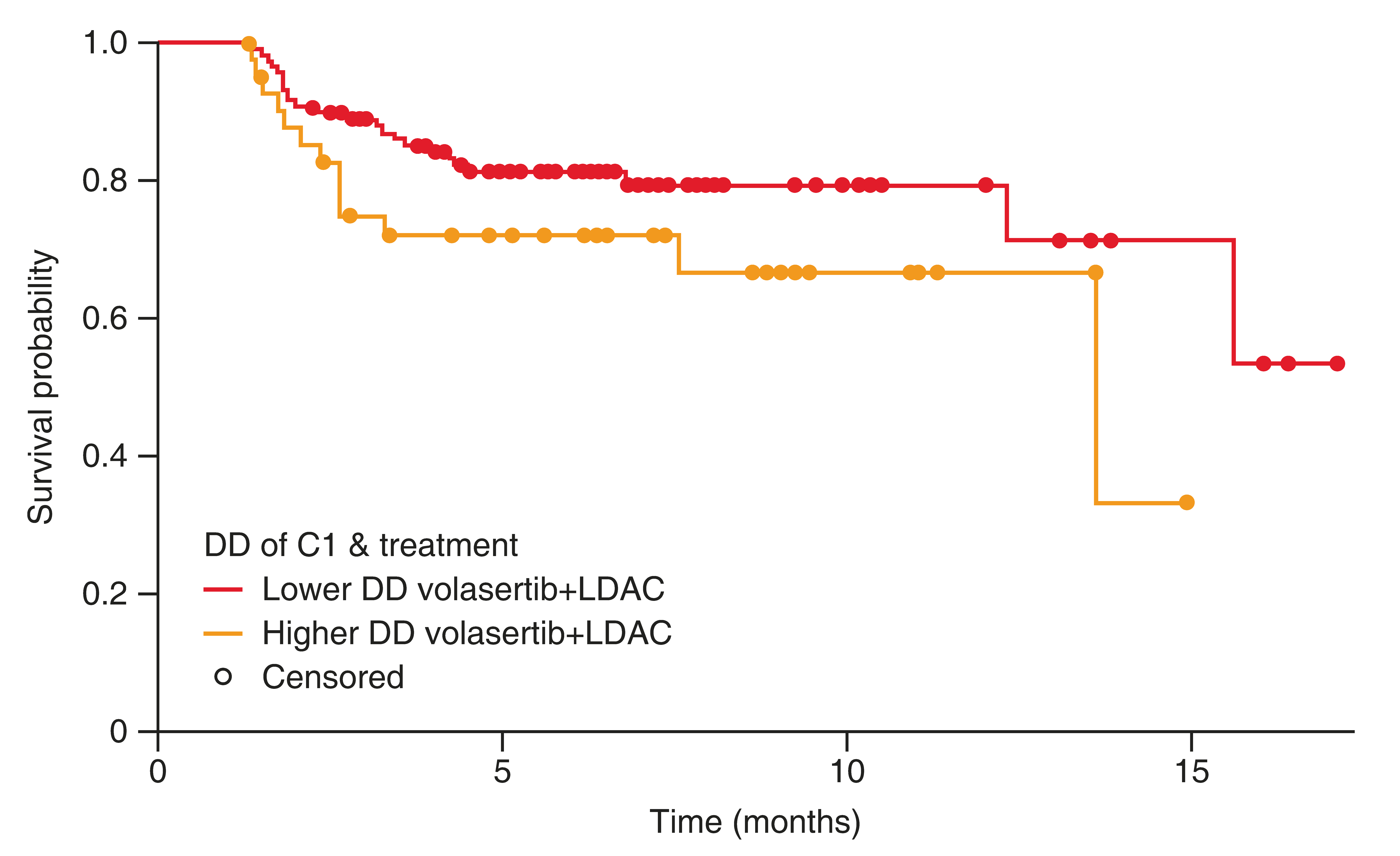
**

**SDC Figure 5. Kaplan–Meier plot of overall survival by dose intensity of V+LDAC in the primary analysis.** C1=cycle 1, DD=dose density, LDAC=low-dose cytarabine.

**
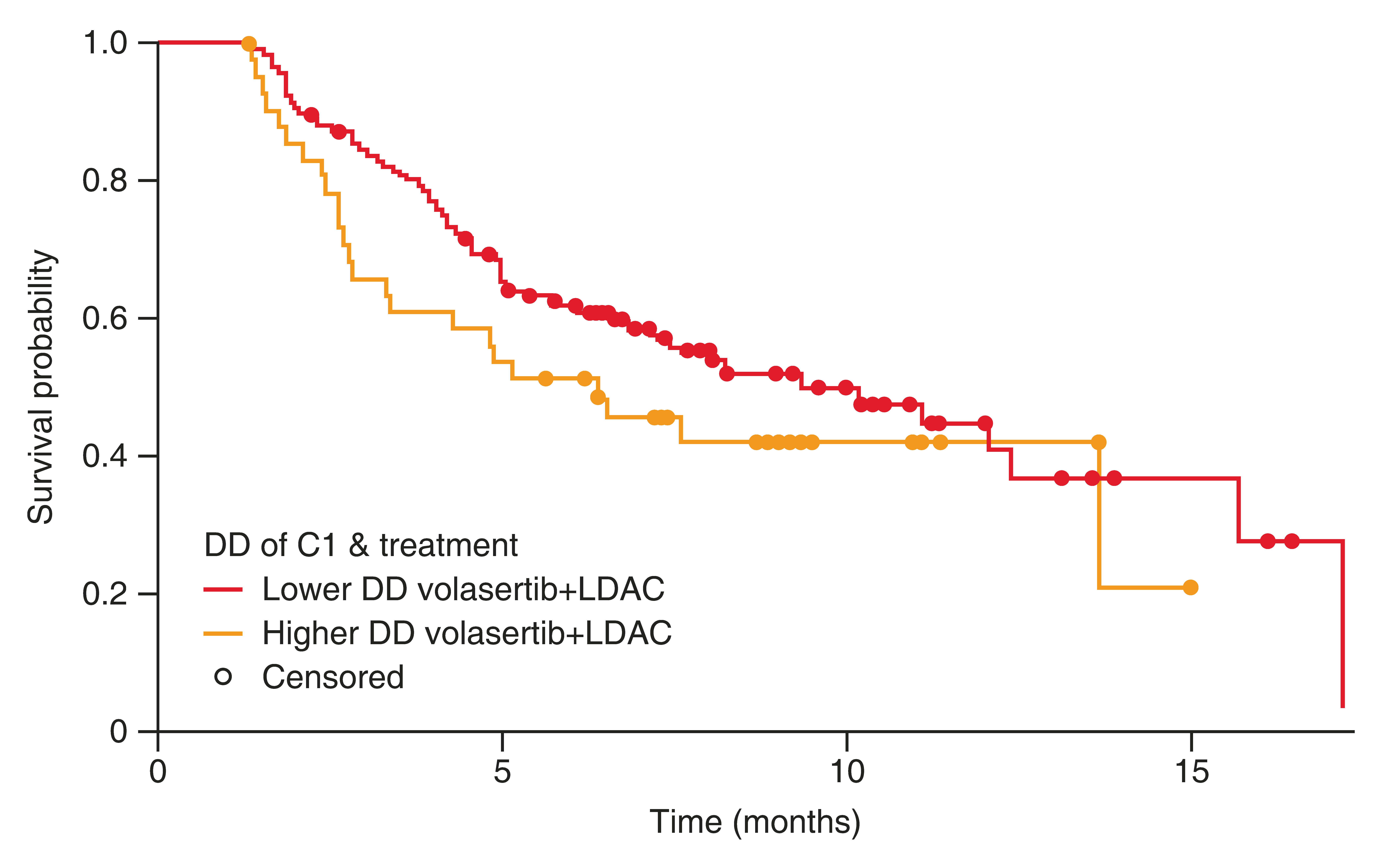
**

**SDC Figure 6. Kaplan–Meier plot, from an unplanned exploratory analysis, of time to fatal infections in patients receiving V+LDAC by duration of prophylactic antibiotic use in the primary analysis set.** The extent of prophylactic antibiotic treatment was calculated as the percentage of days in the risk period (from the first treatment to the last treatment, +21 days) on which prophylactic antibiotics were given: no use, 0%; some use, >0 to ≤60%; frequent use, >60%. Prophylactic antibiotics administered in the V+LDAC arm included fluconazole (36.4%); ciprofloxacin (27.4%); levofloxacin (25.4%); aciclovir (21.2%); ciprofloxacin hydrochloride (16.1%); and vancomycin (11.6%). LDAC=low-dose cytarabine.

**
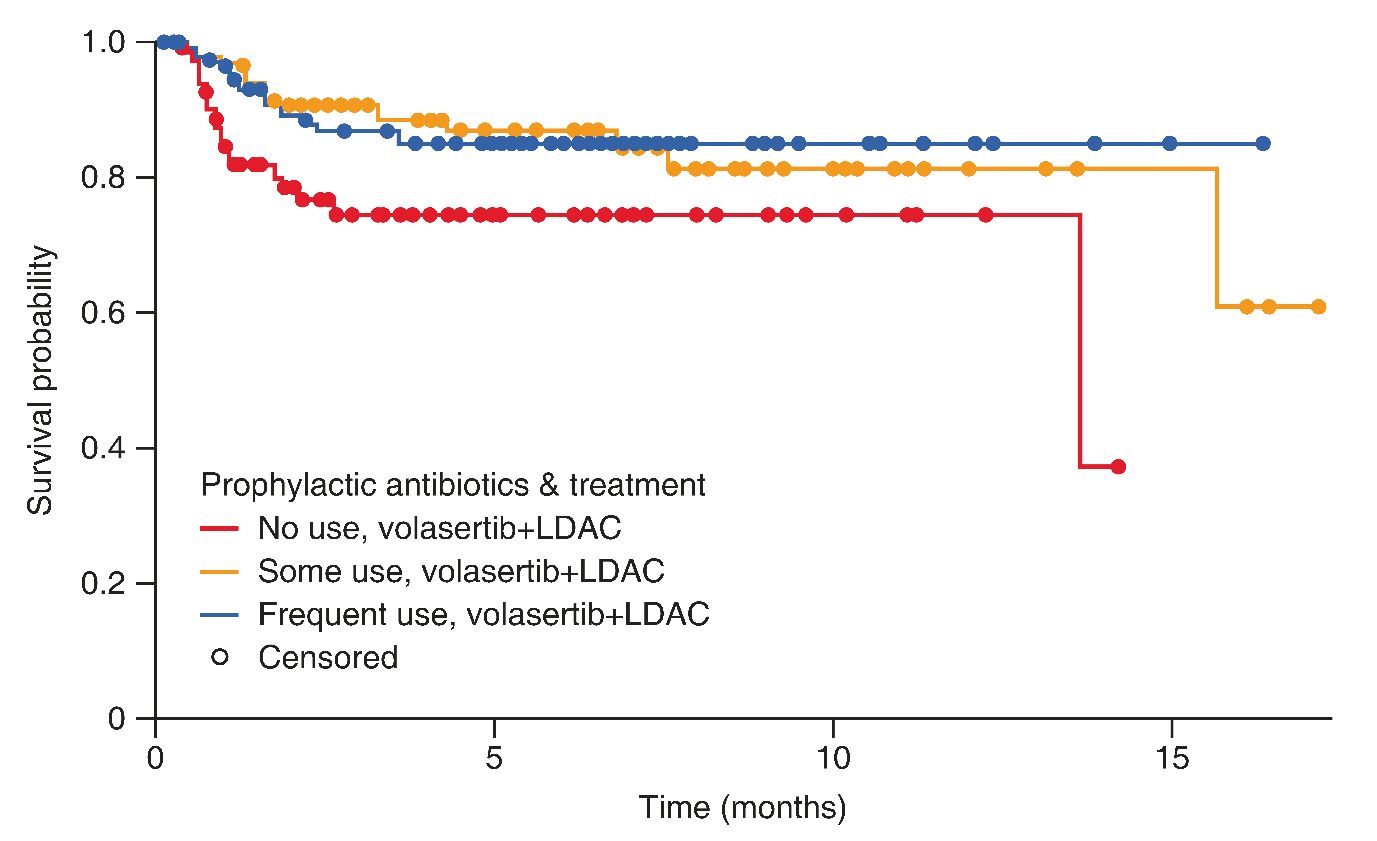
**

**SDC Figure 7. Kaplan–Meier survival plots, from an unplanned exploratory analysis using the cause-specific hazard model for competing risk, of deaths potentially due to lack of treatment efficacy (a) and potentially due to treatment intolerability (b).** CI=confidence interval, LDAC=low-dose cytarabine.


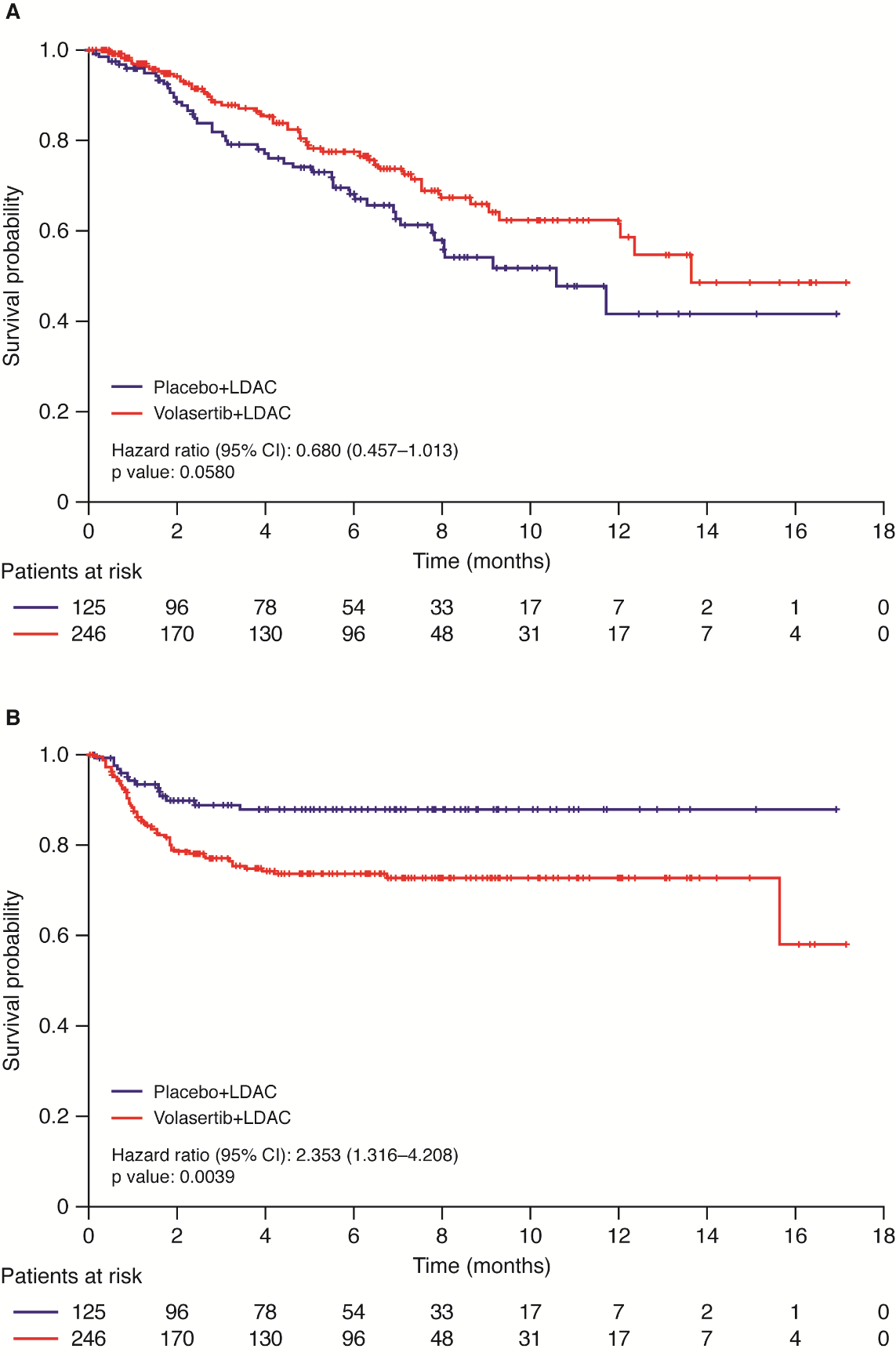


**SDC Figure 8. Cumulative incidence curves of deaths potentially due to lack of treatment efficacy (A) and treatment intolerability (B), from an unplanned exploratory analysis, using the subdistribution model for competing risk.** CI=confidence interval, LDAC=low-dose cytarabine.

**
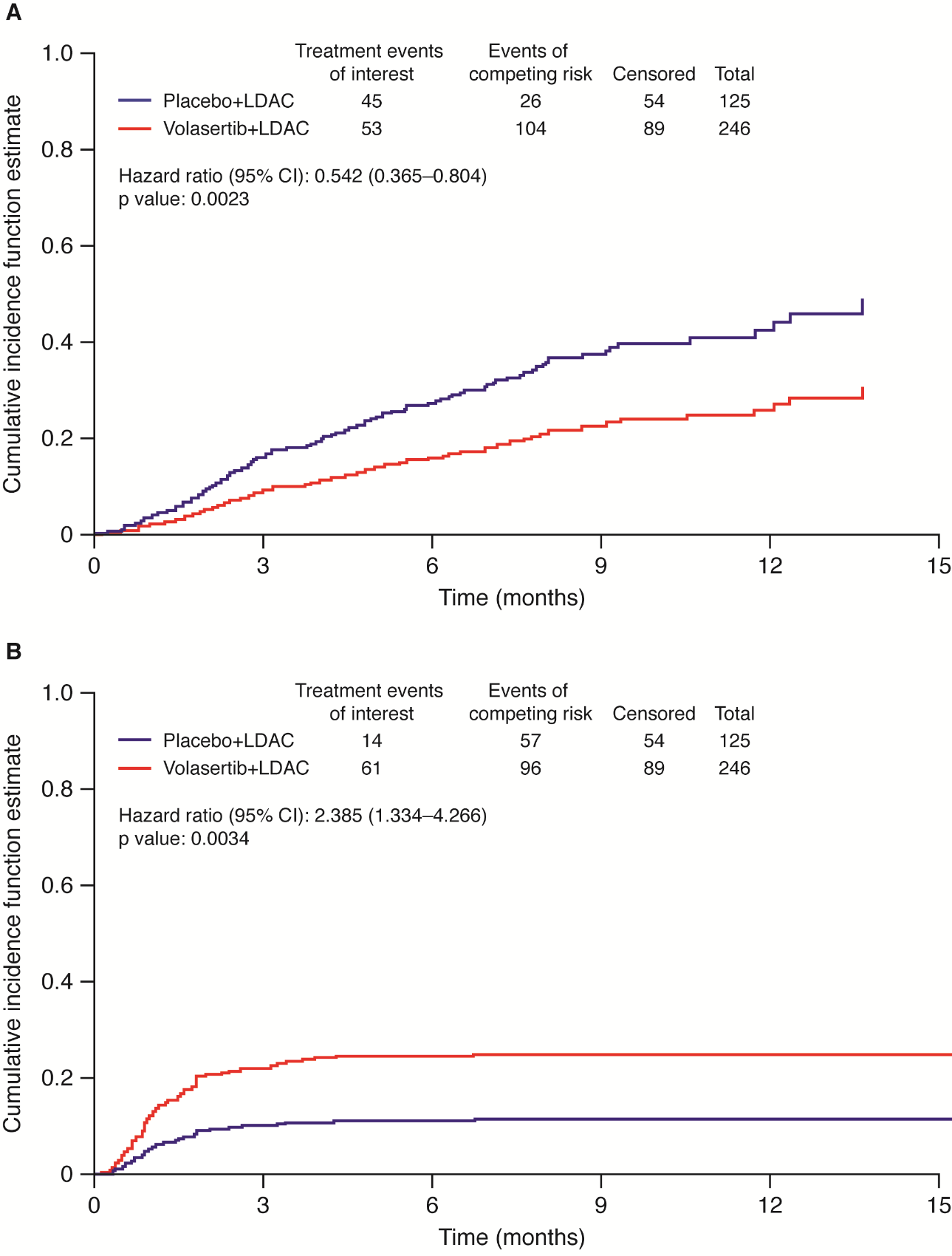
**
